# Re-regeneration to reduce negative effects associated with tail loss in lizards

**DOI:** 10.1038/s41598-019-55231-6

**Published:** 2019-12-10

**Authors:** James I. Barr, Catherine A. Boisvert, Ruchira Somaweera, Kate Trinajstic, Philip W. Bateman

**Affiliations:** 1 School of Molecular and Life Sciences, Curtin University, Kent Street, Bentley, WA 6102 Australia; 2 CSIRO Land and Water, 147 Underwood Avenue, Floreat, WA 6014 Australia; 3 CSIRO Health and Biosecurity, 147 Underwood Avenue, Floreat, WA 6014 Australia

**Keywords:** Ecology, Evolution, Zoology

## Abstract

Many species of lizard use caudal autotomy, the ability to self-amputate a portion of their tail, regenerated over time, as an effective anti-predation mechanism. The importance of this tactic for survival depends on the degree of predation risk. There are, however, negative trade-offs to losing a tail, such as loss of further autotomy opportunities with the regenerated tail vertebrae being replaced by a continuous cartilaginous rod. The common consensus has been that once a tail has been autotomised and regenerated it can only be autotomised proximal to the last vertebral autotomy point, as the cartilage rod lacks autotomy planes. However, anecdotal evidence suggests that although the regenerated portion of the tail is unable to autotomise, it can re-regenerate following a physical shearing event. We assessed re-regeneration in three populations of the King’s skink (*Egernia kingii*), a large lizard endemic to south-west Western Australia and surrounding islands. We show that re-regeneration is present at an average of 17.2% across the three populations, and re-regenerated tissue can comprise up to 23.3% of an individual’s total tail length. The ability to re-regenerate may minimise the costs to an individual’s fitness associated with tail loss, efficiently restoring ecological functions of the tail.

## Introduction

Caudal autotomy is a highly effective anti-predation strategy for squamates, ancestral for all modern taxa and for which we have fossil evidence from Early Permian captorhinids^1,2^. Caudal autotomy, and associated mechanisms, appear to have been lost and re-gained in multiple lizard taxa, depending on the ecological importance of their tail^3–5^. In some species, caudal autotomy is selected against ontogenetically, with fracture planes ossifying as the individuals mature^4,6^. Post-autotomy, an individual’s tail regenerates, with the original bony vertebrae replaced by a rigid cartilage rod that partially ossifies over time^7–9^. Although losing a portion of a tail can have a range of immediate and long term consequences (see^4,10,11^ for reviews), the regenerated tail can restore certain ecological functions associated with the original tail^12–14^.

## Anatomy and Morphology of Caudal Autotomy

There are two ways of shedding a tail: inter-vertebral autotomy, occurring when the tail breaks between inter-vertebral spaces at a point of weakness^4,15^, and intra-vertebral autotomy – the ancestral and more frequent form – occurring at pre-formed breakage planes within a series of caudal vertebrae, termed post-pygal vertebrae^5,6^. Intra-vertebral autotomy is under more complex neurological control of the individual compared to inter-vertebral autotomy, with some species able to autotomise their tail without a physical stimulus^5,16^. The tails of species with intra-vertebral autotomy are constructed as autotomisable segments; however, the cartilage rod that regenerates after autotomy lacks breakage planes and therefore cannot be autotomised, with future autotomy events having occur at the next most proximal vertebrae of the original tail^5,6,9,10,17,18^. In addition to the regenerated tail differing from the original in terms of internal morphology, the external tail in many species show a narrowing at the point of autotomy, as well as changes in scale pattern and colour from the original tail^19^.

## Regeneration After Autotomy Events

It has been assumed that, as the cartilage tube has no breakage planes, lizards cannot autotomise and regenerate sections of already regenerated tails, but must instead autotomise the tail closer to the base each time; e.g. “the regenerated tail … lack[s] intravertebral autotomy fracture planes … and, therefore, subsequent autotomies must take place more proximally”^10^; “[L]izards that experience repeated tail autotomy must lose their tails progressively closer to the tail base …”^17^; “When a tail regenerates, the new portion of is made of a rod of cartilage and thus lacks the intravertebral breakage planes that enable an unregenerated tail to autotomize”^18^ (P 154). However, it may not be as simple as this. Although autotomy and regeneration are primarily, and efficiently, used together, autotomy is not required for caudal regeneration to occur^9,20–22^. Lizards possess the ability to regenerate a cartilage rod and associated tail from an already regenerated portion of their tail, after a shearing event through the cartilage rod, such as a bite from a predator. This regrowth phenomenon, termed re-regeneration, has, as far as we are aware, only been recorded anecdotally^9,23,24^ and may further enhance the capacity of regeneration to reduce negative effects associated with caudal autotomy, such as time and energy trade-offs to growth and reproduction^14,25–27^.

Here we present evidence of re-regeneration in King’s skinks (*Egernia kingii*), a large (up to 244 mm SVL, 550 mm total length) scincid lizard endemic to the south west of Western Australia and its surrounding islands^28,29^. Although juveniles appear to rely more on caudal autotomy than do adults, adults still possess the ability to autotomise their tails^30^. In this study we investigate (1) the occurrence and use of re-regeneration across three isolated populations of *E. kingii* that vary in predation risk, (2) assess the internal morphology of re-regeneration using micro CT technology, and (3) discuss the potential mitigating effects of re-regeneration as well as its use in restoring tail function for lizard ecology.

## Results

**Figure 1:**
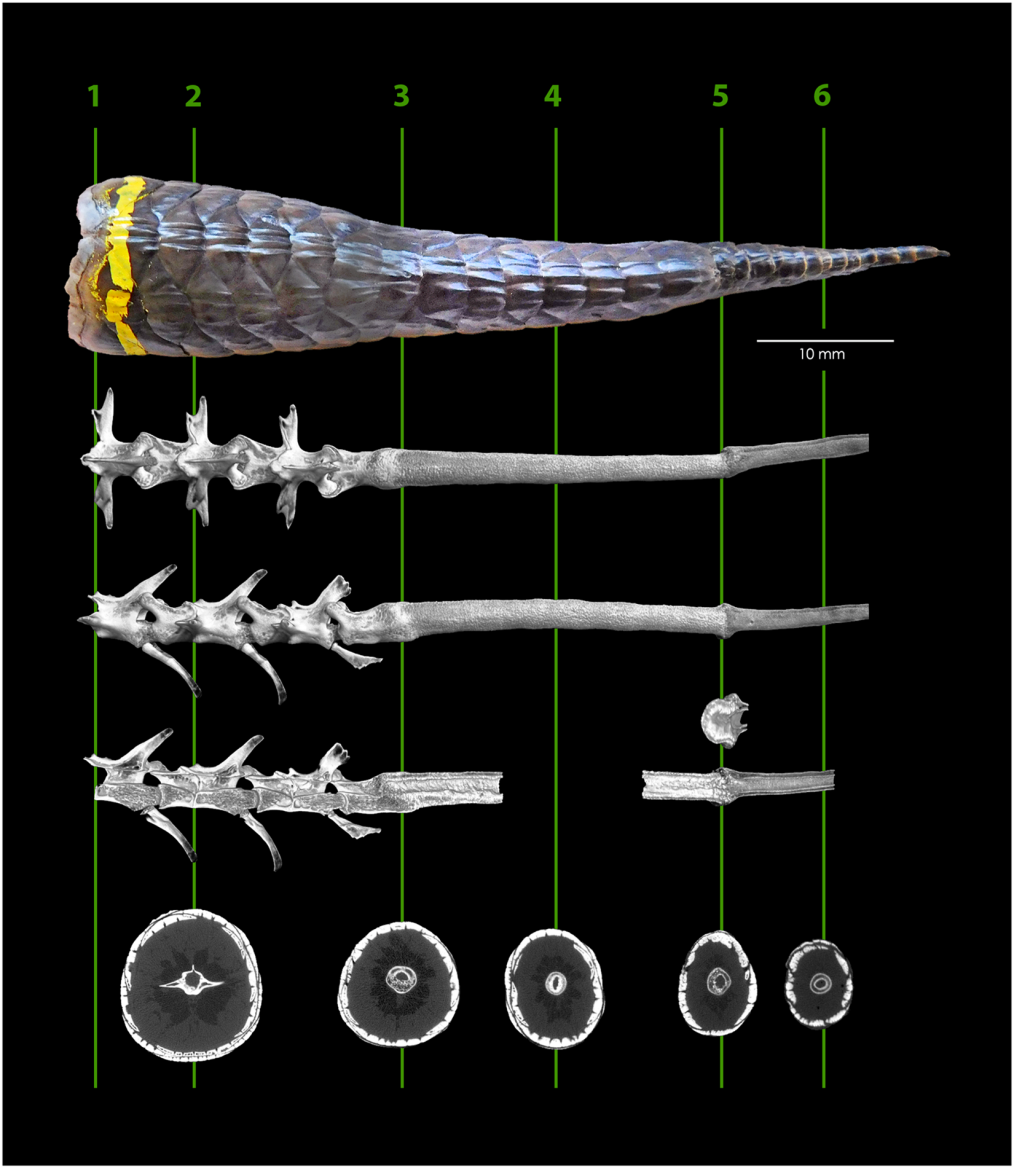
Autotomised tail and 3D model reconstruction from micro CT of *Egernia kingii* showing the fractured vertebrae (1.), two intact vertebrae (2.), vertebrae and primary regeneration fusion point (3.), primary cartilage regeneration (4.), fusion point of primary and secondary cartilage regenerations (5.), and secondary cartilage regeneration (6.) Transverse C.S below correspond to lines on diagram. 1 cm tail tip taken for genetics is missing from the 3D model.

The changes in external morphology associated with regeneration, tail width and scale colour, are evident for both the primary regeneration (Fig. 1, section 3) and the re-regeneration event (Fig. 1, section 5) of the autotomised tail. The CT scan 3D reconstruction of the vertebral column (Fig. 1) shows, from left to right, the distal portion of the fractured (half) vertebrae from the recent autotomy event (1.), two original vertebrae with fracture planes present (2.), the partial vertebrae from the previous, older autotomy event where the cartilage regeneration has been anchored to the vertebrae post-fracture (3.), followed by the older more mature (primary) regenerated tissue (4.), point of secondary regeneration to the primary (5.), and newest (secondary) regenerated tissue (6.), both of which are externally ossified and lack autotomy planes. Coronal and transverse C.S taken from the CT scan highlight the difference in the internal structure of the regenerated tissues, specifically the degree of ossification of the primary regenerated tissue (4.) and secondary regenerated tissue (6.), with the primary regenerated tissue being more ossified than is the secondary regenerated tissue. This is further highlighted by the angled sagittal C.S of the primary and secondary regeneration, with the primary regenerated tissue showing a solid outer sheath, and the secondary regenerated tissue having a distinct outer and inner sheath, with both exhibiting a hollow inner core for the spinal cord tissue (5.).

**Figure 2:**
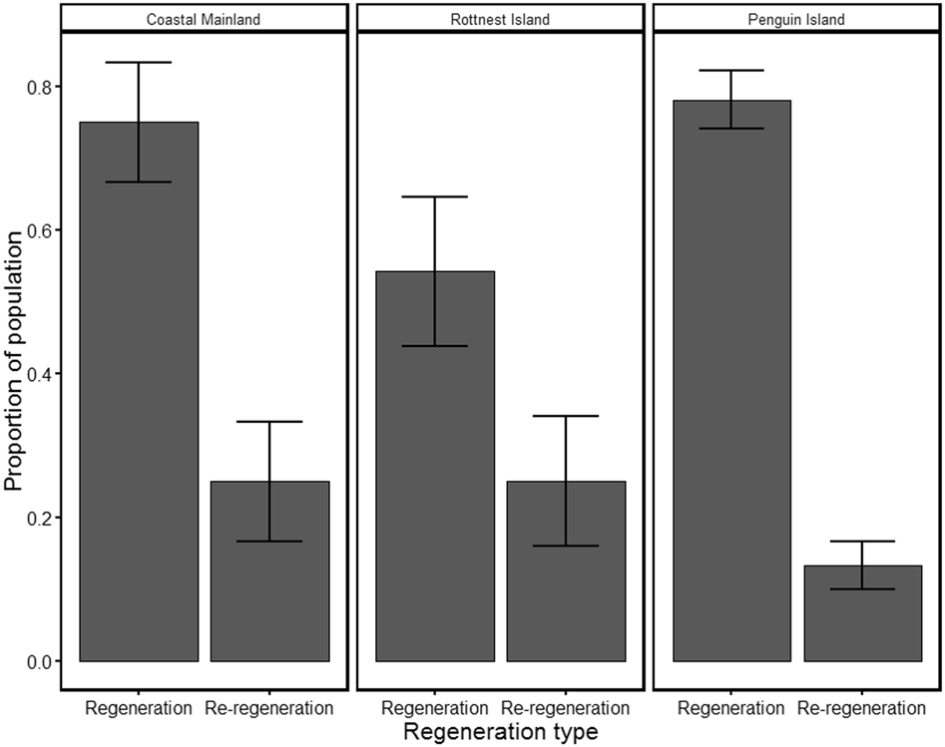
Comparison of proportion of regeneration and re-regeneration of tail tissue for the three study sites from highest predation risk (Coastal Mainland) to lowest predation risk (Penguin Island). Mean ± se are reported.

Field data gathered across the three sites indicated that re-regeneration events were not isolated (Table 1). Higher proportions of re-regeneration were observed in sites with terrestrial predators (Coastal Mainland and Rottnest Island), compared to the site with no terrestrial predators (Penguin Island), with higher levels of overall regeneration observed in the Penguin Island and Coastal Mainland sites (Fig. 2). Re-regeneration events occurred at an average of 17.2% for all individuals captured across the three sites (range 13.3–25.0%), and in 23.5% (range 17.1–46.2%) of individuals that had undergone a regeneration event. Percentage of re-regeneration represented on average (±SD) 18.0 ± 14.8% of the total tail length and 38.5 ± 20.6% of the total regenerated length.

**Table 1:** Summary statistics of *Egernia kingii* populations for the number of individuals caught at each site: those that had regenerated tails and those that had re-regenerated tails; the percentage that the re-regeneration contributed to the total tail (original and regenerated tissue), and the regenerated tissue only.

Metric	All sites	Rottnest Island	Penguin Island	Coastal Mainland
Number caught/ with regeneration/ with re-regeneration	157/115/27	24/13/6	105/82/14	28/20/7
Percentage of total tail length (mean ± SD) that the re-regeneration comprised	18 ± 14.8%	21.2 ± 16.2%	14 ± 11.1%	23.3 ± 19.3%
Percentage of regeneration length (mean ± SD) that the re-regeneration comprised	38.5 ± 20.6%	42.8 ± 18.2%	29.9 ± 18.4%	51.9 ± 20.7%

## Discussion

**Figure 3:**
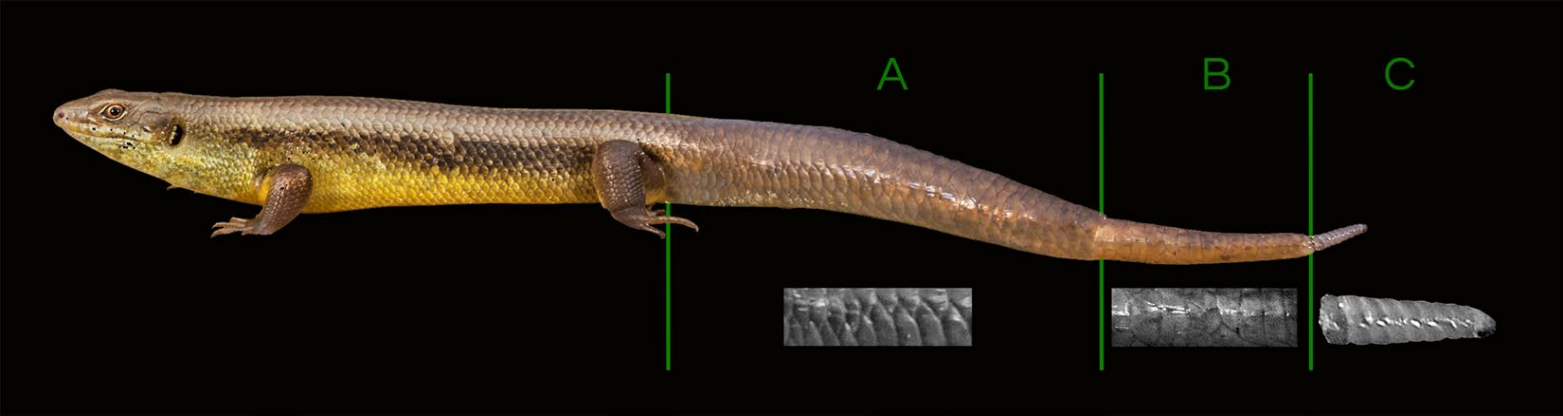
Re-regeneration event in a *Bellatorias major* at Cape York, Queensland, showing the external morphology changes associated with the original (**A**), regenerated (**B**) and re-regenerated (**C**) sections of the tail (photo Ryan Francis).

Losing a tail comes with costs, although these are less severe than being killed by a predator. These costs, whether they be to locomotion^12,31,32^, anti-predation behaviour^33,34^ or even to social status^35^, can be minimised through regeneration of the tail. Here, we have presented unequivocal evidence, through micro CT, that further regeneration of tail tissue is possible if a lizard loses part of the regenerated tail, something has only been anecdotal before^9,23,24^. From field data we show that re-regeneration occurs, and is not an isolated occurrence, in *E. kingii*. Additionally, re-regeneration is known to occur in other species, as seen in *Bellatorias major* (Scincidae) (Fig. 3), a species related to *E. kingii*. The ability for re-regeneration, such as we demonstrate here, is also likely to aid in restoration of certain behavioural and ecological functions of the tail, and subsequently increase fitness and survival.

Lizard taxa that rely heavily on their tail, either as an anti-predation tactic or for locomotion, will incur higher costs for not having a tail^3,11,36,37^, and therefore would be predicted to invest more energy both into tail development^30,38^ and into quicker, and more efficient regeneration^27,39^. Re-regeneration may be more beneficial to populations or species with increased predation risk. Both our sites with terrestrial predators showed higher occurrences of re-regeneration, with the Coastal Mainland site having a higher proportion of re-regenerated tail tissue (Table 1). Additionally, three individuals at the Coastal Mainland site had tertiary regenerations on their tails, indicating further re-regeneration events. Invasive mammals, particularly the European red fox (*Vulpes vulpes*) and feral cat (*Felis catus*) impact on reptile populations in Australia^40^. Our Coastal Mainland site, which is near an urban area, had the highest diversity of predators, including feral cats, dogs, and until recently, red foxes in the area (J. Taylor, pers. comm). As a caveat, intra-specific male-male aggression can also produce high autotomy rates in populations^41,42^. Penguin Island, although lacking terrestrial predators, is known to have high densities of *E. kingii*, and male-male aggression may contribute to the similar regeneration proportion observed in our Coastal Mainland site population (Fig. 2).

Tail regeneration post-autotomy, as well as re-regeneration of the regenerated tissue post-trauma, requires time and energy, and is dependent on other energetic demands that the lizard faces. Recorded rates of caudal regeneration vary considerably between species, ranging from 0.2 mm per day to 2 mm per day^4,43,44^, with some species like *Anniella pulchra* (Anniellidae) regenerating much slower (4.1 mm in 11 months)^45^. As the loss of a tail can have a range of negative effects, it has been proposed that a species will balance the costs of regeneration against requirements for reproductive output^14^. Species that are short lived and mature early will prioritise reproductive output over regeneration, while species that are long lived and mature late, with potential future reproductive seasons will do the opposite^14^. Older individuals of the gecko *Coleonyx variegatus* (Eublapharidae) prioritised energy investment in tail regeneration and less into growth than did younger individuals, which investing more energy in body growth and less in tail regeneration^25^. Furthermore, adult *C. brevis* females prioritise energy into egg production at the expense of tail regeneration^25^.

Re-regeneration is likely to benefit the individual and minimise long term ecological costs associated with caudal autotomy. Firstly, having the ability to regenerate from an already regenerated tail will ensure that an individual does not permanently have a severely reduced tail length following a physical shearing event. Secondly, as a smaller portion of tail would be regenerating, as opposed to if the individual was forced to autotomise a larger portion of tail at the next proximal autotomy plane, both time and energy for regeneration would be reduced. Thirdly, the time an individual would be with a shorter tail would also be reduced. Here, we have presented data on additional regenerative ability in lizards, re-regeneration. We have indicated that, at least in *E. kingii*, this is; 1) not an isolated event and 2) can comprise a large portion of the individual’s tail. Although the regenerated cartilage rod lacks autotomy planes, and its shedding therefore not likely to be under the same conscious control as intra-vertebral autotomy^7–9^, we suggest that re-regeneration may provide an additional component in mitigating the negative effects of caudal autotomy on an individual’s fitness, particularly in populations with high predation risk. Predator size, type and efficiency, i.e. whether attacks tend to be fatal or directed at the tail, may also influence the likelihood or re-regeneration events occurring^46,47^. More research on an ecological comparison of the effects of regeneration and re-regeneration is likely to be fruitful.

## Methods

### Field data

Morphological data for *E. kingii* was collected from three locations along the coast of Western Australia, Rottnest Island (−31.999421°, 115.527540°), Penguin Island (−32.305839°, 115.691340° and Coastal Mainland (−31.868445°, 115.752549°) between 2017 and 2019. General morphologic measurements including snout to vent length (SVL), tail length (TL) and regeneration lengths (RL) were measured to the nearest mm using a plastic ruler. Total regeneration length (length of the whole regenerate) as well as length of individual regeneration segments (primary, secondary or tertiary) were recorded. For analysis, three cases with a tertiary regeneration were included as part of the secondary regeneration length. Percentage of re-regeneration occurrence in populations was established, as well as the percentage of the total tail length comprised of re-regeneration and percentage of total regeneration length comprised of re-regeneration for each individual. All statistics were performed in RStudio Version 1.1.383^48^.

### Re-regeneration specimen and CT analysis

For micro CT a single autotomised tail was collected from an adult (SVL 198 mm) in February 2018 on Rottnest Island, Western Australia. The sample was frozen and then preserved in 100% ethanol after taking a 1 cm tail tip for genetics sampling. The sample was scanned using a micro-CT (SkyScan 1176 scanner; Bruker micro-CT, Kontich, Belgium) at the Centre for Microscopy, Characterisation and Analysis (CMCA), University of Western Australia, Western Australia. The CT scan was performed at 18 μm resolution (50 kV, 500 µA, 390 ms, 0.5 mm Al filter, 0.5° rotation step, 360° scan and two frame averaging) producing 2000 * 1336-pixel images. CT images were reconstructed in NRecon v1.7.1.0 (Bruker micro-CT) using the modified Feldkamp cone- beam algorithm (Gaussian smoothing kernel (2), ring artefact correction (8), beam hardening correction (30%) and threshold for defect pixel masking (3%)). The spinal column was manually selected as a volume of interest (VOI) within CTAnalyser software v1.17.7.2 (Bruker micro-CT). 3D model was recreated in CTvox v3.3.0 r1403 (Bruker micro-CT) and coronal C.S of the model acquired from digital manipulation of the 3D model.

### Ethical statement

All research was carried out in accordance with the Animal Ethics Office of Curtin University (ARE2017-12) and Department of Biodiversity, Conservations and Attractions (DBCA) regulation 17 licence (08-001238-4) for capture and handling of animals.

## Data Availability

The datasets generated during and/or analysed during the current study are available from the corresponding author on reasonable request.
